# Anthropometric Indices and Metabolic Dysfunction–Associated Fatty Liver Disease in Males and Females Living With Severe Obesity

**DOI:** 10.1155/cjgh/5545227

**Published:** 2025-02-16

**Authors:** Fannie Lajeunesse-Trempe, Selena Dugas, Ina Maltais-Payette, Ève-Julie Tremblay, Marie-Eve Piché, Georgios K. Dimitriadis, Annie Lafortune, Simon Marceau, Laurent Biertho, André Tchernof

**Affiliations:** ^1^Department of Specialized Medicine, Internal Medicine, Quebec Heart and Lung Institute, Laval University, Quebec City, Quebec, Canada; ^2^Faculty of Agriculture and Food Sciences, School of Nutrition, Laval University, Quebec City, Quebec, Canada; ^3^Faculty of Life Sciences and Medicine, School of Cardiovascular and Metabolic Medicine & Sciences, King's College London, London, UK; ^4^Department of Medicine, Laval University, Quebec City, Quebec, Canada; ^5^Department of Endocrinology, King's College Hospital NHS Foundation Trust, London, UK; ^6^Department of Surgery, Laval University, Quebec City, Quebec, Canada

**Keywords:** hepatic steatosis, metabolic dysfunction–associated fatty liver diseases, obesity, sex differences

## Abstract

**Introduction:** Metabolic dysfunction–associated fatty liver disease (MAFLD) is highly prevalent among people living with severe obesity (body mass index [BMI] ≥ 35 kg/m^2^). However, it remains unknown how sex and adipose tissue distribution are related to MAFLD onset and progression into metabolic dysfunction–associated steatohepatitis (MASH) or advanced stages of fibrosis.

**Methodology:** We retrospectively studied patients with severe obesity who were eligible for bariatric surgery. Demographic characteristics, biomarkers, and cardiometabolic comorbidities were reported. Anthropometric indices such as BMI, waist circumference (WC), waist-to-hip ratio (WHR), waist-to-height ratio (WHtR), neck circumference (NC), lipid accumulation product (LAP), visceral adiposity index (VAI), body adiposity index (BAI), abdominal volume index (AVI), and body roundness index (BRI) were measured or calculated. MAFLD, MASH, and stages of fibrosis (F1-F4) were established from perioperative liver biopsies. Standardized univariate and multivariate logistic regression analyses were used to examine the association between demographic variables, anthropometric indices, cardiometabolic conditions, and the risk of MASH or severe fibrosis (F2-F4).

**Results:** A total of 2091 participants with severe obesity were included in the analyses; BMI 47.9 ± 7.3 kg/m^2^, age 46.2 ± 11.2 years, and 68.4% females. Overall, MAFLD prevalence was 79.5%, with 44.5% having MASH and 24.4% having severe fibrosis (Stage 2 or higher). No anthropometric indices of adiposity were associated with MASH or fibrosis severity. In this population, female sex was a risk factor for severe fibrosis (OR: 1.27, 95% CI 1.01–1.59, *p* < 0.05).

**Conclusions:** MAFLD and MASH are highly prevalent in individuals living with severe obesity, but no anthropometric indices or laboratory tests are good predictors of MAFLD or MASH in this population. When MAFLD is diagnosed, our results suggest that females with severe obesity might be at higher risk of advanced stages of fibrosis.

## 1. Introduction

In the past decade, major changes in dietary patterns and lifestyle have led to increased prevalence of obesity and cardiometabolic diseases including metabolic dysfunction–associated fatty liver disease (MAFLD). Paralleling the global increase in obesity incidence and prevalence, MAFLD is now the most common cause of liver disease worldwide and its general population prevalence is estimated at 24% [1]. MAFLD remains an underestimated cause of advanced liver diseases such as cirrhosis [2, 3]. Among people living with severe obesity (body mass index [BMI] ≥ 35 kg/m^2^), the prevalence of MAFLD is estimated to be more than 90% in individuals presenting elevated aminotransferases (with and without Type 2 diabetes [T2D]) [4].

MAFLD is a disease spectrum evolving from fatty infiltration in the hepatocytes to hepatic steatosis with inflammation (metabolic dysfunction–associated steatohepatitis [MASH]), progressive fibrosis (commonly graded from F0 to F4), and eventually cirrhosis, all of which in the absence of excessive alcohol intake (less than 20 g a day for women and 30 g a day for men) [5]. More than a quarter of the population diagnosed with MAFLD potentially has MASH, based on elevated hepatic enzymes and no other identifiable cause of liver injury [4]. MASH is a dynamic and reversible metabolic condition in which hepatocyte inflammation can regress to mild degrees of hepatic steatosis, or progress to fibrosis and, in more severe cases (F2 and F3), lead to cirrhosis (F4) [5].

Liver biopsy is the gold standard for diagnosing, quantifying, and characterizing hepatic steatosis and fibrosis, which cannot be implemented routinely due to well-recognized limitations including invasiveness, risk of complications, and cost [6, 7]. Over the past decade, alternative, noninvasive strategies have been suggested to detect MAFLD and MASH, such as serum biomarkers (transaminases, platelets, various metabolites), indexes (fatty liver index [FLI], NAFLD Fibrosis Score, FIB-4) or imaging techniques (ultrasound elastography or magnetic resonance elastography) [7–9]. Yet, biomarkers and imaging techniques have been shown to perform better in individuals with lower BMI [10, 11]. In the case of those affected by severe obesity, screening tools for MAFLD and particularly MASH still need to be tested and validated [12]. Other anthropometric indices such as the lipid accumulation product (LAP) [13], body roundness index (BRI) [14], visceral adiposity index (VAI) [15], abdominal volume index (AVI) [16], or body adiposity index (BAI) [17] have been previously tested for their predictive capacities to detect insulin resistance and the metabolic syndrome [18, 19]. Easy to calculate, these indices have been recently tested for their potential to detect MAFLD (previously defined with NAFLD criteria). Recent evidence suggests that LAP would be the best predictor of MAFLD in Taiwanese men and women [18]. Computed from the third National Health and Nutrition Examination Survey (*N* = 9, 180, statistically weighted to represent 100.05 million US adults), the LAP index has also been suggested to perform better than BMI to identify American adults with higher cardiovascular risk factors including dyslipidemia, high blood pressure, and heart rate [20]. Yet, the predictive value of such indices in individuals with severe obesity needs to be confirmed.

As many other obesity-associated diseases, MAFLD is known to display a major sex difference [1], but recent studies generated conflicting results on the exact impact of sex on MAFLD diagnosis and progression [21, 22]. Current evidence suggests that MAFLD onset and progression are closely associated with regional adipose tissue distribution (i.e., fat preferentially accumulating in the abdominal, visceral compartments in males and postmenopausal females and in the subcutaneous gluteal and femoral region in premenopausal females could be partly involved) [23, 24]. However, it remains unclear if the association between abdominal adiposity and MAFLD or MASH persists in severe obesity [25–30].

The aim of this study was to describe and assess sex differences in the prevalence and histopathological characteristics of MAFLD and MASH, in a large sample of patients with severe obesity. We also explored the association between anthropometric indices and cardiometabolic risk factors with MAFLD, MASH, and severe fibrosis (F2 to F4) in this population.

## 2. Methods

### 2.1. Study Design and Population

This retrospective analysis was conducted using prospectively collected data from a cohort of males and females with a BMI ≥ 35 kg/m^2^, who underwent bariatric surgery with concomitant liver biopsy between January 2013 and July 2018 at the Quebec Heart and Lung Institute-Laval University (IUCPQ-UL) (*n* = 2147). Data were extracted under an IRB-approved protocol (v1.09.06.2023) and prospective electronic database (No. 2024-4043, 22359). Informed consent was obtained before the procedure and data storage. Patient's selection for surgery followed the standard National Institutes of Health recommendations [31]. To be included, participants had to be more than 18 years old and able to provide written consent. Exclusion criteria were chronic liver disease from another etiology than MAFLD such as chronic viral hepatitis, autoimmune hepatitis, drug-induced liver disease, primary biliary cirrhosis, hemochromatosis, Wilson's disease and *α*-1 antitrypsin-deficiency-associated liver disease, and a history of significant alcohol consumption (higher than 20 g a day for women and 30 g a day for men) (*n* = 24) or Type 1 diabetes (*n* = 13). It is noteworthy that when the study was designed in 2018, the newly proposed 2020 MAFLD definition [32] had not been formally acknowledged and widely circulated. Therefore, this study employed the preceding NAFLD criteria [33] and excluded individuals known to have other chronic liver diseases or high levels of alcohol consumption.

### 2.2. Demographic, Anthropometric, and Clinical Variables

Demographic information (age, sex, and tobacco consumption) and anthropometric indices including weight, height, BMI, waist circumference (WC), waist-to-hip ratio (WHR), waist-to-height ratio (WHtR), and neck circumference (NC) were assessed preoperatively. Obesity-associated comorbidities (obstructive sleep apnea [OSA], hypertension, coronary artery disease [CAD], dyslipidemia, and T2D) were reported based on standardized definitions [34], or when it was mentioned as a diagnosis in the patient's medical record.

Biomarkers considered were aminotransferases including alanine aminotransferase (ALT), *γ*-glutamyltransferase (GGT), alkaline phosphatase (ALP), bilirubin, platelet count, International National Ratio (INR), albumin, fasting blood lipid levels (low-density lipoprotein cholesterol [LDL-C], high-density lipoprotein cholesterol [HDL-C], total cholesterol [TC], apolipoprotein-B [Apo-B], triglycerides [TGs]), glycosylated hemoglobin A1c (HbA1c), and ferritin. Nonfasting blood tests and laboratory analyses were done within 24 h before liver biopsy, at the time of hospital admission, using standardized biochemical analysis and performed either in our institution or in provincial laboratories. The FLI score was calculated using the Bedogni formula [35].

### 2.3. Anthropometric Indices

Adiposity-related anthropometric indices scores were calculated according to the following formulas:

(1) $$\begin{array}{c} \text{LAP}\left(\text{male}\right)=\text{WC}\left(\text{cm}\right)–65\times \text{TG}\left(\frac{\text{mmol}}{L}\right)\left[13\right],\\ \text{LAP}\left(\text{female}\right)=\text{WC}\left(\text{cm}\right)-58\times \text{TG}\left(\frac{\text{mmol}}{L}\right)\text{in females}\left[13\right],\\ \text{VAI}\left(\text{male}\right)=\left(\frac{\left(\text{WC}\left(\text{cm}\right)\right)}{\left(39.68+1.88x\text{BMI}\right)}\right)x\left(\frac{\text{TG}\left(\text{mmol}/L\right)}{1.03}\right)x\left(\frac{1.31}{\text{HDL}\left(\text{mmol}/L\right)}\right)\left[15\right],\\ \text{VAI}\left(\text{female}\right)=\left(\frac{\left(\text{WC}\left(\text{cm}\right)\right)}{\left(39.58+1.89x\text{BMI}\right)}\right)x\left(\frac{\text{TG}\left(\text{mmol}/L\right)}{0.81}\right)x\left(\frac{1.52}{\text{HDL}\left(\text{mmol}/L\right)}\right)\left[15\right],\\ \text{BAI}\left(\text{both sexes}\right)=\frac{\text{hip circumference}\left(\text{cm}\right)}{\text{height}{\left(m\right)}^{3/2}}–18\left[17\right],\\ \text{AVI}\left(\text{both sexes}\right)=\frac{2\;x\;{\text{WC}\left(\text{cm}\right)}^{2}+0.7\;x\;{\left(\text{WC}\left(\text{cm}\right)-\text{hip cirumference }\left(\text{cm}\right)\right)}^{2}}{1000}\left[16\right],\\ \text{BRI}\left(\text{both sexes}\right)=364.2–365.5x\sqrt{1-{\left(\frac{\left(\text{WC }\left(m\right)/2\times \pi \right)}{\left(0.5\;x\text{ height }\left(m\right)\right)}\right)}^{2}}\left[14\right]. \end{array}$$

### 2.4. MAFLD and MASH Diagnosis

Liver biopsies were performed during bariatric procedures. All biopsy specimens were taken immediately as wedges from the edge of the left liver lobe, fixed in formalin, and stained with hematoxylin/eosin and Masson's trichrome. The histological characteristics, MAFLD, and MASH diagnosis were classified according to the Brunt grading system by a trained pathologist [36], which has been validated elsewhere [37]. MAFLD was successfully assessed for 2091 patients, and MASH was diagnosed and characterized by pathological features including percentage of steatosis, ballooning, and lobular and periportal inflammation as well as the level of fibrosis (F0 to F4). Severe fibrosis was characterized per definition as F2 or more [37]. In a small number of participants (*n* = 19), histopathological characteristics could not be interpreted due to technical issues with the specimen or because the liver biopsy could not be performed during surgery.

### 2.5. Statistical Analysis

An *a priori* power analysis was conducted using *G*^∗^ Power Version 3.1 for sample size estimation, based on data from previous studies, which looked at the association between features of obesity (BMI and WC) and MAFLD [38]. All analyses were performed using Stata 15.0 software. All continuous variables were normally distributed. Mean values, percentage, and standard deviations (SD) were used to describe population characteristics. Categorical variables were expressed using percentages. Sex differences in patient characteristics, and diagnosis were assessed using *t*-test and chi-squared test. To study the association between demographics, anthropometric indices, biomarkers, and cardiometabolic comorbidities with MAFLD, MASH, and severe fibrosis (F2 to F4), standardized odds ratios (OR) were calculated using univariate logistic regression and multivariate logistic regression adjusted for sex, diagnosis of T2D, and BMI for predictor variables WC or WHR specifically, given the previously estimated collinearity. Two-sided *p* < 0.05 was considered statistically significant.

## 3. Results

A total of 2091 participants with a BMI ≥ 35 kg/m^2^ met the inclusion criteria, but a complete dataset was available from a total of 2082 participants (631 males [30.6%] and 1431 females [69.4%], *p* < 0.05). As shown in Table 1, the mean age of participants was 48 ± 11 years for males and 44 ± 10 years for females. The mean BMI was 48.3 ± 8.3 kg/m^2^ in males and 47.7 ± 6.7 kg/m^2^ in females (*p* = 0.7). For a similar BMI, males presented a significantly higher WC compared to females (144.8 ± 15.0 vs. 132.5 ± 13.1 cm, respectively), higher WHR (1.06 ± 0.11 vs. 0.93 ± 0.09), and higher NC (49.9 ± 6.1 vs. 43.1 ± 4.1 cm) (*p* < 0.05). Males also had higher LAP, AVI, and BRI compared to females, but the latter presented higher levels of VAI and BAI. Males were also more likely to have obesity-associated cardiometabolic diseases (hypertension, CAD, dyslipidemia, and T2D) but not OSA.

Subgroup analyses were conducted specifically among individuals diagnosed with MAFLD (Supporting Table 1) and MASH (Supporting Table 2), stratified by gender. Generally, men diagnosed with MAFLD or MASH exhibited comparable anthropometric indices when compared to the overall cohort. However, in women diagnosed with MASH, WC was significantly elevated compared to their counterparts with or without MAFLD (*p* < 0.05). Prevalence rates of hypertension, dyslipidemia, and T2D were notably higher in both men and women affected by MAFLD and MASH in comparison with the overall cohort.

Remarkably, among men diagnosed with both MAFLD and MASH, the prevalence of CAD was twice as high (10.1% and 11.5%, respectively, vs. 5.1% in the general cohort). Men with MASH exhibited significantly higher rates of hypertension and T2D compared to the overall cohort (80.4% vs. 64.2% for hypertension and 88.9% vs. 67.7% for T2D, *p* < 0.05). Additionally, it was observed that all men diagnosed with MASH concurrently experienced OSA.

Table 2 shows MAFLD and MASH prevalence, as well as histopathological characteristics by sex, according to the Brunt Grading system [36]. Almost all patients, males and females, had at least 5% of steatosis reported on their liver biopsy, reaching MAFLD definition [6] (81.6% of males and 80.2% of females, *p* = 0.3). When diagnosed with MAFLD, more than half of these participants presented distinct features of MASH (46.9% in males and 44.2% in females, *p* = 0.3). In general, both sexes had a similar level of detrimental histopathological characteristics. A total of 145 (23.0%) males presented features of severe fibrosis (F2 or higher) on their biopsy sample compared to 366 (25.6%) females (*p* = 0.07). The proportion of males and females diagnosed with cirrhosis (F4) was also similar (1.7% of males vs. 2.7% of females, *p* = 0.2).

The association between demographic characteristics, anthropometric indices, biomarkers, cardiometabolic conditions, and MAFLD or MASH is presented in Supporting Table 3 and Table 3, respectively. In individuals with severe obesity, age, sex, and smoking status were not associated with a higher risk of developing MASH. Commonly used anthropometric indices BMI, WC, WHR, WHtR, and NC were not associated with histopathologically defined MASH. Some calculated anthropometric scores such as AVI, BAI, and BRI presented a statistically significant association with MASH, although their estimated OR were relatively modest (Table 3). Among all obesity-associated cardiometabolic comorbidities, only T2D significantly increased the risk of MASH (OR: 1.27, 95% CI 1.04–1.57, *p* < 0.05).

Examining the association between these characteristics and the degree of fibrosis severity, our analyses did not show a significant relationship with age, smoking status, measurements of body fat distribution (including calculated anthropometric scores), or any of the comorbidities (Table 4). Only male sex was negatively associated with an increased risk of developing severe hepatic fibrosis (OR: 0.79, 95% CI 0.63–0.99). Predictive capacities of the FLI score to detect biopsy-diagnosed MAFLD in people with severe obesity are presented in Supporting Table 4. The FLI score, which is based on BMI and WC as well as GGT and TG [35], was not predictive of MAFLD in people with a BMI ≥ 35 kg/m^2^.

## 4. Discussion

This study reports the results of the largest sample using a comprehensive dataset including demographics, anthropometric indices (adipose tissue distribution scores), biomarkers, and cardiometabolic conditions as predictors of biopsy-assessed MAFLD, MASH, and severe hepatic fibrosis in severe obesity. Our results show that in individuals with a BMI ≥ 35 kg/m^2^, anthropometric indices (weight, BMI, WC, WHR, and WHtR) are associated with neither MAFLD and MASH nor severe fibrosis. Calculated anthropometric indexes (LAP, VAI, AVI, BAI, and BRI) are not correlated with MAFLD and severe fibrosis. Even though AVI, BAI, and BRI show a statistically significant relationship with MASH, the estimated OR are relatively modest. Consequently, we cannot confidently conclude on a significant clinical association.

These results cannot be attributed to a Type 2 error due to lack of statistical power. Indeed, our power analyses show that using a significance criterion of *α* = 0.05 and power = 0.80, the minimum sample size needed with this effect size is *N* = 191 for multivariate logistic regression. The obtained sample size of *N* = 631 in males and *N* = 1431 in females is therefore adequate to test the study hypothesis. Surprisingly, despite being younger, having lower measurements of abdominal adiposity (WC, WHR, and WHtR) and suffering less from cardiometabolic conditions (T2D, CAD, hypertension, and dyslipidemia), females with severe obesity present similar detrimental histopathological features of MAFLD and a similar risk of MASH and advanced stages of fibrosis compared to males. In this specific population, female sex is even significantly associated with an increased risk of severe hepatic fibrosis.

MAFLD detection and management are challenging, mainly because MAFLD remains asymptomatic and undetectable using conventional blood tests for a long period, during which the condition evolves and can become terminal. Gold-standard liver biopsy comes with many limitations, including risk of severe complications [6, 7]. Imaging techniques such as MRI or ultrasound elastography have not been validated in populations with severe obesity [10], can be expensive, and are not easy to access in all clinical settings. Anthropometric measurements of adiposity are known determinants of cardiometabolic disorders. Previous cross-sectional studies have shown that measurements of total adiposity and adipose tissue distribution (BMI and WC) are significantly associated with MAFLD and MASH in males and females with overweight or moderate obesity [39, 40]. However, whether anthropometric measurements can predict MAFLD, MASH, and hepatic fibrosis in severe obesity had not been elucidated yet.

As mentioned, our analyses do not show significant associations between anthropometric indices of adiposity and histopathological MAFLD, MASH, or fibrosis. Among risk factors for MASH, only T2D shows a significant association (OR: 1.27, 95% IC 1.04–1.57, *p* < 0.05). This is consistent with previous studies reporting on the association between anthropometric indices of adiposity and cardiometabolic disorders in people with severe obesity [29, 38], but not all [30, 41]. In 2007, Drapeau et al. have shown that in pre- and postmenopausal females with a BMI ≥ 40 kg/m^2^ (mean age of 35.1 ± 0.5 years, mean BMI of 53.4 ± 0.7 kg/m^2^, and mean WC of 138.4 ± 1.2 cm), WC was not associated with detrimental metabolic traits (impaired fasting glucose, plasma HDL-C levels, and TC/HDL-C ratio) [41]. It is well established that increased visceral adipose tissue (VAT) is associated with cardiometabolic risk factors and recent studies suggest that abdominal subcutaneous adipose tissue (SAT) does not correlate with MAFLD [34, 35]. Our results suggest that in the context of severe obesity, anthropometric indices of abdominal adiposity, including WC and WHR, might not reflect increased intra-abdominal VAT accumulation and rather represent an indicator of a high level of SAT.

Ooi et al. have recently published a prospective study including 216 participants who had bariatric surgery (mean BMI 45.9 ± 8.9 kg/m^2^, age 44.4 ± 12.1 years, 75.5% female) and have shown that MASH and fibrosis (diagnosed using intraoperative liver biopsies and classified according to NAFLD activity score [NAS]) increase independently of BMI categories (OR 2.28–3.46, all *p* < 0.05) [33]. However, despite a population similar to ours in terms of age, BMI, and sex, the authors report on a surprisingly lower rate of MASH and severe fibrosis (12.0% and 5.1%), as well as a lower rate of T2D (26.0%), dyslipidemia (20.1%), hypertension (46.0%), and OSA (29.6%) [42]. This suggests that within people living with severe obesity, cardiometabolic conditions including MASH and advanced stages of liver fibrosis might progress differently and might develop at different timepoints in relation to obesity onset, based on individual underlying pathophysiological mechanisms which are not completely understood.

Among other factors, genetics, ethnicity, diet, microbiota, cardiorespiratory fitness, and level of physical activity have shown to have an independent and significant impact on the risk of developing MAFLD and MASH [4, 43–46]. Moreover, an increasing body of evidence suggests that obesity duration might also have a significant impact on cardiometabolic health (insulin resistance, T2D, dyslipidemia, and cardiovascular disease) [47]. Unfortunately, within our study analysis, inquiries regarding the duration of obesity were not conducted, and genetic examination was not accessible. More studies, including genetic testing and polygenic risk score elaboration, are needed to elucidate the complex relationship between adipose tissue distribution and cardiometabolic disorders, specifically MAFLD and MASH, in this specific population.

Our results from this specific population do not show sex differences in the prevalence of MAFLD, MASH, and detrimental histopathological features of MAFLD including advanced stages of fibrosis. Surprisingly, even with a younger age (mean age of 44.0 ± 10.0 years old), a lower proportion of obesity-associated comorbidities (T2D, dyslipidemia, hypertension, and CAD), and lower anthropometric measurements of the upper body (NC) and abdominal obesity (WC and WHR), females present a similar prevalence of MAFLD, MASH, and severe fibrosis compared to males. Similarly, in a recent study using the FIB-4 index (platelet count × √ALT) to estimate liver fibrosis in 6967 participants from Japan (48% male, mean age of 50 ± 15 years old and mean BMI of 22.3 ± 4.2 kg/m^2^), Tanaka et al. reported that male sex and BMI are negatively associated with estimated hepatic fibrosis (hazard ratio [HR] of 0.77, 0.88, *p* value of 0.042, 0.001), but not WC (HR of 1.03, *p* value of 0.012) [15]. Menopausal status was not available in our study, but our results are also consistent with a large study from Guerrero-Romero and Rodríguez-Morán. Combining data from 3 large American cohorts and including a total of 1112 patients with biopsy-proven MAFLD show that premenopausal females, but not males and postmenopausal females, are more at risk of lobular inflammation, hepatocyte ballooning, and Mallory-Denk bodies compared to males and postmenopausal females [16]. However, their results also show that males and postmenopausal females might be more affected by hepatic fibrosis than premenopausal females [16]. The results we report on could be explained by abdominal adipose tissue distribution, which seems to manifest differently among males and females diagnosed with severe obesity. It is commonly accepted that females preferentially accumulate adipose tissue in the gluteofemoral regions (hips and thighs) [48]. However, this phenotype may be less distinctive in females with higher BMI values. In our sample, females present high mean WHR and WHtR (0.93 ± 0.091 and 0.82 ± 0.086) reflecting a clear propensity to store adipose tissue in the abdominal region, even when compared to other similar populations [30, 49]. Females also present higher levels of VAI, a calculated proxy for VAT function and insulin resistance [18], despite a lower AVI, which estimates the overall abdominal volume. It might be possible that in our specific population, females accumulate more VAT, which translates into a higher risk of detrimental histopathological features of MAFLD and MASH. Menopausal status and hormonal factors including an androgenic fat distribution signature often found in women living with severe obesity and/or with PCOS [50, 51] might be other contributing factors. Undoubtedly, scientific literature shows that sex partly modulates MAFLD progression, from hepatic steatosis to MASH, fibrosis, and eventually cirrhosis. Further research incorporating abdominal imagery techniques such as computed tomography scans or magnetic resonance is needed.

In terms of limitations, the reported results need to be considered according to the reliability of physical measurements, in cases of severe obesity. WC, hip circumference, and even NC measurements are, at times, difficult to perform. This is indeed underlined in our population by the large intervals we obtained measuring WC (from 112 to 220 cm), WHR (from 0.71 to 2.85), and NC (from 30 to 104). However, it was decided to include all reasonable values as they likely represent the variability of such measurements in real-life clinical settings. Moreover, certain common independent variables like AST or fasting glucose were not included in the baseline blood tests conducted prior to bariatric surgery, making it impossible to retrieve them retrospectively from medical records. Consequently, some other validated indexes such as the TG glucose-WC or TG glucose-WHR [52] could not be tested in the context of this study. Our population included twice as many females as males, and although this is a representative ratio of surgical bariatric populations worldwide, this can also partially impair the external validity of this study. Important factors (genetic testing, diet, fitness, level of physical activity, or obesity duration, among others) were not included in this study and will need to be considered in future work on MAFLD in people living with severe obesity. Antihyperglycemic medications are not reported in this study. Considering the high numbers of our participants diagnosed with T2D (54.0% with 16.8% being on oral therapy) and given the known effect of certain drugs on MAFLD and MASH, i.e., thiazolidinediones (TZDs), sodium glucose cotransporter-2 (SGLT-2) inhibitors, and glucagon-like peptide-1 (GLP-1) receptor agonists [53], this might impact the associations reported here. Given that this study was designed in 2018, the exclusion criteria adhered to previous NAFLD guidelines, which advocated excluding alternative liver disease causes and defining “excessive” alcohol consumption [33]. However, the updated 2020 International MAFLD Guidelines incorporate alcohol consumption within the diagnostic framework [32]. Consequently, 24 participants were excluded based on the former criteria, and their data could not be retrospectively retrieved. As they constituted only 1.1% of the total population, we assume that their exclusion has minimal effects on the overall conclusions of this study.

In conclusion, MAFLD and MASH are highly prevalent in individuals living with severe obesity. When MAFLD is diagnosed, males and females are both at risk of being characterized by detrimental histopathological features of MASH, but our results suggest that females with severe obesity might be more at risk of advanced stages of fibrosis. In cases of severe obesity, anthropometric features and indexes of obesity cannot detect MAFLD, MASH, and severe hepatic fibrosis. Given the high prevalence and the reversibility of MAFLD and MASH in this population, more studies examining sex differences in MAFLD and including measurements of VAT, SAT, or other biological features are needed.

## Figures and Tables

**Table 1:** Baseline characteristics.

Baseline characteristics	Male	Female	*p* value	Both sexes
*N* (%)	631 (30.6)	1431 (69.4)	< 0.05	2062 (100.0)
Age, years (±SD)	48.0 (11.0)	44.0 (10.0)	< 0.05	46.2 (11.2)
Smoking status *n* (%)	51 (8.1)	165 (11.5)	< 0.05	216 (10.4)
Weight, kg (±SD)	146.7 (27.0)	124.6 (19.8)	< 0.05	131.5 (24.5)
Body mass index, kg/m^2^ (±SD)	48.3 (8.3)	47.7 (6.7)	0.07	47.8 (7.4)
Waist circumference, cm (±SD)	144.8 (15.0)	132.5 (13.1)	< 0.05	134.2 (21.5)
Waist-to-hip ratio (±SD)	1.1 (0.1)	0.9 (0.09)	< 0.05	0.97 (0.1)
Waist-to-height ratio (±SD)	0.8 (0.08)	0.8 (0.1)	< 0.05	0.8 (0.1)
Neck circumference, cm (±SD)	49.9 (6.1)	43.1 (4.1)	< 0.05	45.3 (5.9)
Comorbidities				
Hypertension, *n* (%)	405 (64.2)	624 (43.6)	< 0.05	1029 (49.9)
Coronary atherosclerosis diseases, *n* (%)	32 (5.1)	56 (3.9)	< 0.05	88 (4.3)
Dyslipidemia, *n* (%)	318 (50.4)	416 (29.1)	< 0.05	734 (35.6)
Type 2 diabetes, *n* (%)	427 (67.7)	703 (49.1)	< 0.05	1130 (54.8)
Treated with insulin therapy	81 (19.0)	78 (11.1)		159 (7.7)
Treated with antihyperglycemic medication only	152 (36.0)	199 (28.3)		351 (17.0)
On diet only	123 (28.8)	275 (39.1)		398 (19.3)
Not known	71 (16.6)	151 (21.5)		222 (10.8)
Obstructive sleep apnea, *n* (%)	495 (40.5)	727 (59.5)	< 0.05	1222 (59.2)
Laboratories				
Alanine aminotransferase ALT, IU/L (±SD)	29.4 (17.4)	31.6 (21.7)	< 0.05	30.8 (21.2)
*γ*-Glutamyltransferase, IU/L (±SD)	38.5 (33.9)	42.0 (47.7)	0.1	39.9 (41.3)
Alkaline phosphatase, IU/L (±SD)	77.1 (20.5)	78.5 (22.9)	0.2	77.5 (21.3)
Bilirubin, *μ*mol/L (±SD)	8.3 (5.4)	7.9 (3.6)	0.1	8.0 (4.3)
Albumin, g/L (±SD)	41.9 (2.7)	42.0 (2.8)	0.6	42.0 (2.8)
International National Ratio, (±SD)	1.5 (11.2)	1.1 (2.4)	0.2	1.3 (7.2)
Platelets, ⁣^∗^ 10^9^/L (±SD)	247.4 (60.5)	248.4 (62.0)	0.7	248.5 (60.9)
Ferritin, *μ*g/L (±SD)	133.6 (116.6)	147.6 (157.2)	< 0.05	144.5 (149.5)
HbA1c, % (±SD)	6.1 (1.1)	6.2 (1.1)	0.3	6.2 (1.1)
LCL cholesterol, mmol/L (±SD)	2.5 (0.8)	2.6 (1.4)	0.07	2.5 (1.3)
HDL cholesterol, mmol/L (±SD)	1.2 (0.4)	1.2 (0.3)	0.8	1.2 (0.3)
Total cholesterol, mmol/L (±SD)	5.3 (2.6)	4.6 (4.0)	0.3	4.9 (13.7)
Triglycerides, mmol/L (±SD)	1.7 (1.0)	1.7 (0.9)	0.7	1.7 (0.9)
Apo (B), g/L (±SD)	0.9 (0.2)	0.9 (0.3)	0.07	0.9 (0.2)
Fatty liver index score (±SD)	71.1 (24.7)	62.2 (26.4)	< 0.05	64.4 (26.5)
Anthropometric indexes				
Lipid accumulation product	135.9 (3.5)	126.4 (1.8)	< 0.05	116.9 (1.7)
Visceral adiposity index	2.3 (0.7)	3.0 (0.06)	< 0.05	2.8 (0.05)
Abdominal volume index	43.1 (0.3)	36.2 (0.2)	< 0.05	38.3 (0.2)
Body adiposity index	45.0 (0.3)	46.7 (0.2)	< 0.05	46.2 (0.2)
Body roundness index	11.8 (0.1)	11.6 (0.07)	< 0.05	11.7 (0.06)

**Table 2:** MAFLD prevalence, MASH prevalence, and histopathological characteristics among sex.

Histopathological characteristics	Males (*n* = 631)	Females (*n* = 1431)	*p* value
MAFLD *n* (%)	515 (81.6)	1148 (80.2)	0.3
MASH *n* (%)	296 (46.9)	632 (44.2)	0.3
Percentage of steatosis^1^%	28.4 (24.7)	26.9 (23.8)	< 0.05
Lobular inflammation *n* (%)			
Grade 0	262 (41.5)	619 (43.3)	0.6
Grade 1	212 (33.6)	439 (30.7)	
Grade 2	32 (5.1)	69 (4.8)	
Grade 3	14 (2.2)	24 (1.7)	
Hepatocellular ballooning n (%)			
Grade 0	399 (63.2)	896 (62.6)	0.7
Grade 1	109 (17.3)	239 (16.7)	
Grade 2	11 (1.7)	16 (1.1)	
Portal tract inflammation n (%)			
Grade 0	114 (18.1)	268 (18.7)	0.9
Grade 1	354 (56.1)	761(53.2)	
Grade 2	48 (7.6)	115 (8.4)	
Grade 3	4 (0.6)	7 (0.5)	
Fibrosis *n* (%)			
Grade 0	188 (29.8)	389 (27.2)	0.4
Grade 1	189 (30.0)	396 (27.7)	
Grade 2	87 (13.8)	215 (15.0)	
Grade 3	47 (7.4)	112 (7.8)	
Grade 4	11 (1.7)	39 (2.7)	

^1^Results expressed as mean (SD).

**Table 3:** Association between demographic characteristics, anthropometric measurements, comorbidities, and MASH using standardized OR from univariate and multivariate logistic regression.

Variables	OR	95% CI	*p* value
Age	0.99	0.98–1.01	0.5
Sex (male)	1.04	0.84–1.28	0.7
Smoking status	0.99	0.72–1.35	0.9
Weight^1^	0.98	0.88–1.09	0.6
Body mass index^1^	0.92	0.84–1.02	0.1
Waist circumference^2^	1.10	0.94–1.30	0.2
Waist-to-hip ratio^2^	1.04	0.93–1.17	0.5
Waist-to-height ratio^2^	1.01	0.86–1.17	1.0
Neck circumference	0.90	0.79–1.04	0.2
Type 2 diabetes^3^	1.27	1.04–1.57	< 0.05
Dyslipidemia^1^	1.06	0.85–1.32	0.6
Hypertension^1^	0.86	0.69–1.08	0.1
CAD1	1.07	0.65–1.78	0.8
Obstructive sleep apnea^1^	0.61	0.14–2.50	0.5
Lipid accumulation product	1.0009	0.99–1.00	0.2
Visceral adiposity index	0.99	0.95–1.04	0.9
Abdominal volume index	1.03	1.02–1.04	< 0.05
Body adiposity index	1.02	1.01–1.04	< 0.05
Body roundness index	1.08	1.04–1.11	< 0.05

Abbreviation: MASH, metabolic-associated steatohepatitis.

^1^Multivariate logistic regression adjusted for sex and Type 2 diabetes.

^2^Multivariate logistic regression adjusted for sex, Type 2 diabetes, and BMI.

^3^Multivariate logistic regression adjusted for sex.

**Table 4:** Association between demographic characteristics, anthropometric measurements, comorbidities, and severe hepatic fibrosis using standardized OR from multivariate logistic regression.

Variables	OR	95% CI	*p* value
Age	1.01	0.99–1.01	0.4
Sex (male)	0.79	0.63–0.99	< 0.05
Smoking status	1.30	0.94–1.79	0.1
Weight^1^	0.94	0.84–1.07	0.3
Body mass index^1^	0.93	0.84–1.04	0.2
Waist circumference^2^	0.91	0.76–1.07	0.3
Waist-to-hip ratio^2^	0.99	0.85–1.10	0.6
Waist-to-height ratio^2^	0.88	0.75–1.04	0.1
Neck circumference^1^	0.95	0.82–1.11	0.5
Type 2 diabetes^3^	1.09	0.87–1.35	0.4
Dyslipidemia^1^	0.99	0.79–1.25	0.9
Hypertension^1^	0.95	0.77–1.18	0.7
CAD^1^	1.37	0.81–2.30	0.2
Obstructive sleep apnea^1^	1.08	0.87–1.35	0.5
Lipid accumulation product	1.0007	0.99–1.002	0.3
Visceral adiposity index	1.02	0.97–1.07	0.5
Abdominal volume index	1.006	0.99–1.02	0.4
Body adiposity index	1.009	0.99–1.03	0.2
Body roundness index	1.03	0.99–1.06	0.2

^1^Multivariate logistic regression adjusted for sex and Type 2 diabetes.

^2^Multivariate logistic regression adjusted for sex, Type 2 diabetes, and BMI.

^3^Multivariate logistic regression adjusted for sex.

## Data Availability

The data used to support the findings of this study are restricted by the Quebec Heart and Lung Institute-Laval University (IUCPQ-UL) research ethical committee regulation in order to protect patient privacy. Data are available from the corresponding author (Laurent Biertho [laurentbiertho@gmail.com]) for researchers who meet the criteria for access to confidential data.

## Supporting Information

Supporting Information Table 1: Descriptive Characteristics of men and women diagnosed with MAFLD.

Supporting Information Table 2: Descriptive Characteristics of men and women diagnosed with MASH.

Supporting Information Table 3: Association between demographic characteristics, anthropometric measurements, comorbidities, and MAFLD using standardized OR from multivariate logistic regression.

Supporting Information Table 4: MAFLD prevalence among groups based on suggested FLI cutoffs.
