# Supplementary material for: Anthropometric Indices and Metabolic Dysfunction–Associated Fatty Liver Disease in Males and Females Living With Severe Obesity
Source: Can J Gastroenterol Hepatol. 2025 Feb 16;2025:5545227. doi: 10.1155/cjgh/5545227 (PMC11847611; doi:10.1155/cjgh/5545227)
Supplement: Supporting Information — Additional supporting information can be found online in the Supporting Information section. [file 5545227.f1.docx]

**Supplementary Table 1**

**Descriptive characteristics of men and women diagnosed with MAFLD**

| **Baseline Characteristics** | **Men**  **n=515** | | **Women n=1148** | | **p-value** |
| --- | --- | --- | --- | --- | --- |
| Age, years (± SD) | 48.7 | 10.9 | 44.8 | 11.3 | <0.05 |
| Smoking status n (%) | 47 | 9.1 | 152 | 13.2 | <0.05 |
| Weight, kg (± SD) | 146.8 | 26.8 | 124.7 | 19.8 | <0.05 |
| Body mass index, kg/m^2^ (± SD) | 48.3 | 8.1 | 47.7 | 6.8 | <0.05 |
| Waist circumference, cm (± SD) | 144.6 | 14.8 | 132.5 | 13.2 | <0.05 |
| Waist-to-hip ratio ( ± SD) | 1.1 | 0.1 | 0.9 | 0.1 | <0.05 |
| Waist-to-height ratio (± SD) | 0.8 | 0.1 | 0.8 | 0.1 | 0.4 |
| Neck circumference, cm (± SD) | 43.1 | 4.2 | 49.9 | 6.1 | <0.05 |
| **Comorbidities** |  |  |  |  |  |
| Hypertension, n (%) | 381 | 73.9 | 558 | 48.6 | <0.05 |
| Coronary Atherosclerosis Diseases, n (%) | 52 | 10.1 | 27 | 2.3 | <0.05 |
| Dyslipidemia, n (%) | 304 | 59.0 | 383 | 33.4 | <0.05 |
| Type 2 diabetes, n (%) | 399 | 77.4 | 645 | 56.2 | <0.05 |
| Obstructive Sleep Apnea, n (%) | 463 | 90.0 | 664 | 57.8 | <0.05 |
| Lipid Accumulation Product | 135.1 | 3.7 | 125.8 | 2.0 | <0.05 |
| Visceral Adiposity Index | 2.3 | 0.8 | 3.0 | 0.06 | <0.05 |
| Abdominal Volume Index | 43.0 | 0.3 | 36.2 | 0.2 | <0.05 |
| Body Adiposity Index | 45.0 | 0.3 | 46.7 | 0.2 | <0.05 |
| Body roundness Index | 11.8 | 0.1 | 11.5 | 0.08 | <0.05 |

MAFLD: Metabolic Dysfunction-Associated Fatty Liver Disease

**Supplementary Table 2**

**Descriptive characteristics of men and women diagnosed with MASH**

| **Baseline Characteristics** | **Men**  **n=296** | | **Women n=632** | | **p-value** |
| --- | --- | --- | --- | --- | --- |
| Age, years (± SD) | 48.6 | 10.9 | 45.9 | 10.6 | <0.05 |
| Smoking status n (%) | 33 | 11.1 | 81 | 12.8 | <0.05 |
| Weight, kg (± SD) | 148.3 | 19.8 | 126.0 | 27.0 | <0.05 |
| Body mass index, kg/m^2^ (± SD) | 48.6 | 6.7 | 48.3 | 8.2 | <0.05 |
| Waist circumference, cm (± SD) | 134.7 | 13.1 | 145.7 | 14.8 | <0.05 |
| Waist-to-hip ratio ( ± SD) | 1.1 | 0.1 | 0.9 | 0.1 | <0.05 |
| Waist-to-height ratio (± SD) | 0.8 | 0.1 | 0.8 | 0.1 | 0.7 |
| Neck circumference, cm (± SD) | 50.1 | 5.3 | 43.7 | 4.1 | <0.05 |
| **Comorbidities** |  |  |  |  |  |
| Hypertension, n (%) | 238 | 80.4 | 313 | 49.5 | <0.05 |
| Coronary Atherosclerosis Diseases, n (%) | 34 | 11.5 | 11 | 1.7 | <0.05 |
| Dyslipidemia, n (%) | 195 | 65.9 | 220 | 34.8 | <0.05 |
| Type 2 diabetes, n (%) | 263 | 88.9 | 408 | 64.6 | <0.05 |
| Obstructive Sleep Apnea, n (%) | 296 | 100 | 379 | 60.0 | <0.05 |
| Lipid Accumulation Product | 138.8 | 4.1 | 129.6 | 2.8 | 0.05 |
| Visceral Adiposity Index | 2.3 | 0.09 | 3.0 | 0.08 | 0.06 |
| Abdominal Volume Index | 45.6 | 0.5 | 37.3 | 0.3 | <0.05 |
| Body Adiposity Index | 47.3 | 0.4 | 48.0 | 0.3 | <0.05 |
| Body roundness Index | 12.0 | 0.2 | 12.1 | 0.1 | 0.7 |

MASH: Metabolic Dysfunction-Associated Steatohepatitis

**Supplementary Table 3**

**Association between demographic characteristics, anthropometric measurements, comorbidities and MAFLD using standardized OR from multivariate logistic regression**

| **Variable** | **OR** | **05% IC** | **p-value** |
| --- | --- | --- | --- |
| Age | 0.99 | 0.98-1.010 | 0.7 |
| Sex (male) | 1.25 | 0.91-1.72 | 0.2 |
| Smoking status | 1.22 | 0.75-1.99 | 0.4 |
| Weight^1^ | 1.05 | 0.89-1.23 | 0.6 |
| Body mass index^1^ | 1.06 | 0.91-1.23 | 0.4 |
| Waist circumference^2^ | 0.92 | 0.73-1.15 | 0.5 |
| Waist-to-hip ratio^2^ | 1.09 | 0.90-1.31 | 0.4 |
| Waist-to-height ratio^2^ | 0.94 | 0.76-1.16 | 0.6 |
| Neck circumference^1^ | 1.19 | 0.95-1.49 | 0.1 |
| Type 2 diabetes ^3^ | 0.81 | 0.60-1.09 | 0.2 |
| Dyslipidemia^1^ | 0.87 | 0.64-1.19 | 0.4 |
| Hypertension^1^ | 0.88 | 0.65-1.18 | 0.4 |
| CAD^1^ | 0.58 | 0.31-1.07 | 0.09 |
| Obstructive Sleep Apnea | 1.22 | 0.2 | 0.91-1.65 |
| Fatty liver index | 0.99 | 0.99-1.00 | 0.3 |
| Lipid Accumulation Product | 0.99 | 0.99 – 1.00 | 0.2 |
| Visceral Adiposity Index | 0.98 | 0.91 – 1.04 | 0.4 |
| Abdominal Volume Index | 0.99 | 0.97 – 1.01 | 0.4 |
| Body Adiposity Index | 0.99 | 0.97 – 1.01 | 0.3 |
| Body roundness Index | 0.97 | 0.92 – 1.03 | 0.3 |

^1^ Multivariate logistic regression adjusted for sex and type 2 diabetes

^2^ Multivariate logistic regression adjusted for sex, type 2 diabetes and BMI

^3^ Multivariate logistic regression adjusted for sex

MAFLD: Metabolic Dysfunction-Associated Fatty Liver Disease

**Supplementary Table 4**

**MAFLD prevalence among groups based on suggested FLI cutoffs**

|  | **Normal liver**  **n (%)** | **MAFLD**  **n (%)** |
| --- | --- | --- |
| FLI < 30 | 20 (7.6) | 242 (92.4) |
| FLI ≥ 30, <60 | 80 (16.2) | 413 (83.8) |
| FLI ≥ 60 | 145 (10.9) | 1191 (89.3) |

FLI: Fatty liver index

MAFLD: Metabolic-Associated Fatty Liver Disease

Between-group differences were all significant (p<0.05)
